# Screening Active Components from Yu-Ping-Feng-San for Regulating Initiative Key Factors in Allergic Sensitization

**DOI:** 10.1371/journal.pone.0107279

**Published:** 2014-09-08

**Authors:** Dandan Shen, Xuejian Xie, Zhijie Zhu, Xi Yu, Hailiang Liu, Huizhu Wang, Hongwei Fan, Dawei Wang, Guorong Jiang, Min Hong

**Affiliations:** 1 National Standard Laboratory of Pharmacology for Chinese Materia Medica, Jiangsu Key Laboratory for Pharmacology and Safety Evaluation of Chinese Materia Medica, Nanjing University of Chinese Medicine, Nanjing, China; 2 Nanjing General Hospital of Nanjing Military Command, Nanjing, China; 3 Department of Clinical Pharmacology Lab, Nanjing First Hospital, Nanjing Medical University, JiangSu Nanjing, China; 4 Suzhou Hospital of Tradional Chinese Medicine, Suzhou, China; 5 Jiangsu Academy of Traditional Chinese Medicine, Nanjing, China; 6 Pharmacy Department, The First People's Hospital of Taicang, Taicang, China; Fundação Oswaldo Cruz, Brazil

## Abstract

Yu-ping-feng-san (YPFS) is a Chinese medical formula that is used clinically for allergic diseases and characterized by reducing allergy relapse. Our previous studies demonstrated that YPFS efficiently inhibited T helper 2 cytokines in allergic inflammation. The underlying mechanisms of action of YPFS and its effective components remain unclear. In this study, it was shown that YPFS significantly inhibited production of thymic stromal lymphopoietin (TSLP), an epithelial cell-derived initiative factor in allergic inflammation, *in vitro* and *in vivo*. A method of human bronchial epithelial cell (16HBE) binding combined with HPLC-MS (named 16HBE-HPLC-MS) was established to explore potential active components of YPFS. The following five components bound to 16HBE cells: calycosin-7-glucoside, ononin, claycosin, sec-o-glucosylhamaudol and formononetin. Serum from YPFS-treated mice was analyzed and three major components were detected claycosin, formononetin and cimifugin. Among these, claycosin and formononetin were detected by 16HBE-HPLC-MS and in the serum of YPFS-treated mice. Claycosin and formononetin decreased the level of TSLP markedly at the initial stage of allergic inflammation *in vivo*. Nuclear factor (NF)-κB, a key transcription factor in TSLP production, was also inhibited by claycosin and formononetin, either in terms of transcriptional activation or its nuclear translocation *in vitro*. Allergic inflammation was reduced by claycosin and formononetin when they are administered only at the initial stage in a murine model of atopic contact dermatitis. Thus, epithelial cell binding combined with HPLC-MS is a valid method for screening active components from complex mixtures of Chinese medicine. It was demonstrated that the compounds screened from YPFS significantly attenuated allergic inflammation probably by reducing TSLP production via regulating NF-κB activation.

## Introduction

Yu-ping-feng-san (YPFS) is a well-known Chinese prescription that consists of *Radix Astragali* (RA, Huangqi), *Rhizoma Atractylodis Macrocephalae* (RAM, Baizhu), and *Radix Saposhnikoviae* (RS, Fangfeng). Clinically, YPFS is mainly used in allergic diseases, such as allergic rhinitis [1], urticaria [2], [3] and asthma [4], [5], especially in reducing the relapse rate and disease severity. Recent studies have indicated that YPFS affects expression of T cell receptor (TCR) and major histocompatibility complex (MHC) class II expression on CD4^+^ T cells in a mouse model of asthma, regulates the balance of T helper(Th)1/Th2 cells in murine allergic airway disease [6], and decreases interleukin(IL)-17 level [7]. Our previous studies have demonstrated that YPFS significantly inhibits Th2-cell-mediated atopic contact dermatitis (ACD) [8] and ovalbumin (OVA)-induced allergic asthma. YPFS markedly decreased the IL-4 level and consequently increased the ratio of interferon-γ/IL-4 [9]. However, these studies were focused on the inflammatory phase of allergy, which does not explain the mechanisms underlying the effect of YPFS on relapse.

The pharmacological effects of some components of YPFS have been reported. For example, flavonoids show antioxidant [10] and antiviral [11] activity. Total saponin in RA promotes antibody production and immune responses [12], [13]. Chromone glucosides in RS and atractylenolide I and III are anti-inflammatory components [14]. Polysaccharide in these herbs has an effect on the immune system [15]–[17]. However, these components have no direct activity against relapse of allergic diseases.

Most allergic diseases are mediated by Th2 lymphocytes. Researchers have come to realize that epithelial cells (ECs) play a critical role in stimulating and regulating local immune responses [18]. Studies of thymic stromal lymphopoietin (TSLP) derived from ECs have provided important evidence that ECs can regulate the immune response to initiate the allergic response. TSLP mRNA is highly expressed in human primary skin keratinocytes and bronchial ECs [18], [19]. TSLP contributes directly to the activation of dendritic cells (DCs), which then migrate into the lymph nodes and prime allergen-specific Th2 responses [20]. Therefore, TSLP might be a master switch for allergic inflammation at the EC–DC interface [21]. It was presumed that the key mechanism involved in the reduction of allergy recurrence by YPFS might be related to regulation of TSLP derived from ECs. Therefore, we examined the effect of YPFS on TSLP production in the present study. ECs were utilized to screen for potential active components in YPFS, and their effects on TSLP production and allergic inflammation were determined.

We selected human bronchial epithelial cells (16HBE cells) to screen for potential active components in YPFS effective on ECs. This method combined 16HBE cell binding with HPLC-MS, therefore it was called 16HBE-HPLC-MS. Previously, our group has successfully established some cell-binding methods to screen for potential active components in Chinese medicine, including hepatocyte [22], [23], erythrocyte [24], macrophage [25] and splenocyte [26] binding. By utilizing these methods, some components from Chinese medicine were identified and demonstrated to be active. The components in serum of mice treated with YPFS were analyzed to determine whether they were absorbed into the blood. The effects of these detected components on allergic inflammation and TSLP production were evaluated *in vitro* and *in vivo*.

## Methods

### Materials

Radix Astragali (RA; Inner Mongolia, China), Rhizoma Atractylodis Macrocephalae (RAM; Zhejiang, China), and Radix Saposhnikoviae (RS; Heilongjiang, China) were purchased from the Herbal Decoction Slices division of Nanjing Pharmaceutical Company (Nanjing, China). Botanic identification was confirmed by Professor Chungen Wang (Nanjing University of Chinese Medicine, China). Calycosin-7-glucoside, ononin, claycosin, sec-o-glucosylhamaudol and formononetin were purchased from Tianjin Marker Bio-Tech Co. Ltd (Tianjin, China). Cimifugin was purchased from National Institutes For Food And Drug Control (Beijing, China).

### Animals and cells

All procedures involving animals were approved by the Animal Care and Use Committee of Nanjing University of Chinese Medicine and strictly performed according to the Guide for the Care and Use of Laboratory Animals. BALB/c mice were purchased from Beijing Military Medical Academy and Shanghai Slac Laboratory Animal Company. All animals were maintained at Nanjing University of Chinese Medicine under specific pathogen-free conditions at 18°C–25°C and 50%–60% humidity, and were used at 6–10 weeks of age. 16HBE cells were purchased from the Cell Bank of the Chinese Academy of Medical Sciences (Beijing, China) and cultured in RPMI 1640 medium (Wisent, Saint-Jean-Baptiste, QC, Canada) supplemented with 10% fetal bovine serum (Wisent) at 37°C and 5% CO_2_.

### Preparation of YPFS extracts

Five hundred grams of YPFS (3∶1∶1) were immersed in 3.25 L ethanol:water (95∶5, v/v) for 1 h and then refluxed for 2 h. The extraction process was repeated twice and the extracts were combined, filtered, and evaporated to dryness using a vacuum concentrator system (CH-9230; BUCHI Labortechnik, Flawil, Switzerland) at 60°C. The extract was then subjected to analysis at a concentration of 1.5 g crude drug/g extract.

### TSLP production model *in vivo*

BALB/c mice were treated with 0.6% fluorescein isothiocyanate (FITC; Sigma, St. Louis, MO, USA) in 20 µL acetone and dibutyl phthalate (1∶1, vehicle) on both ears on day 1 and 2, and sacrificed on day 3. Mice were treated once daily with YPFS or formononetin and claycosin 2 days before treatment with FITC until day 3 of the model. Both ears were removed and ground into homogenates with ice–phosphate–buffered saline (PBS), and the homogenates were centrifuged at 4000 g at 4°C for 15 min. The supernatant was stored at −80°C before analysis. The concentration of TSLP in ear homogenate was analyzed by mouse TSLP ELISA kits (eBioscience, San Diego, CA) according to the manufacturer's instructions. Total protein level in the homogenates was examined by BCA kit (Jiancheng, Nanjing, China). TSLP level was assessed with the formula: concentration of TSLP in the homogenate/total protein (pg/mg).

### Measurement of TSLP production induced by tumor necrosis factor-alpha (TNF-α) in 16HBE cells *in vitro*

16HBE cells were seeded into 96-well plates at a density of 8×10^5^cells/mL and incubated at 37°C under 5% CO_2_. At 80%–90% confluency, cells were stimulated with TNF-α (1.25 µg/mL; PeproTech, Rocky Hill, NJ, USA) and 4%–10% serum from YPFS-treated mice simultaneously for 12 h. The concentration of TSLP in culture supernatant was detected by Human TSLP ELISA kit (eBioscience) according to the manufacturer's instructions.

### 16HBE cell binding assay

16HBE cell suspensions (8×10^6^cells/mL) were incubated with YPFS extract at a concentration of 0.1 g/mL with gentle shaking at 37°C for 1 h and then centrifuged at 1100 g for 5 min. Cell pellets were washed three times with D-Hank's Solution to remove unbound components, followed by centrifugation at 200 g for 5 min. The final washing eluate was collected as one of the controls. Cells were incubated with 8 mL hydrochloride acid D-Hanks' (pH 4.0) for 1 h at 37°C, followed by centrifugation at 400 g for 5 min to liberate the components bound to the cells. The supernatant was collected by centrifugation and was referred to as the desorption eluate. The blank desorption eluate, in which YPFS extract was replaced by RPMI 1640, was generated using the method described above.

The desorption solution or the final washing eluate (2 mL) was separately mixed with ethyl acetate (4 mL), vortexed for 3 min, and centrifuged at 1600 g for 5 min. Supernatants were collected and dried under nitrogen at 45°C. Residues were dissolved with 300 μL methanol and centrifuged at 10000 g for 5 min and filtered by 0.45 μm nylon membrane filter before HPLC-MS analysis.

### Preparation of serum from YPFS-treated mice

Serum from YPFS-treated mice was prepared as described previously [27]. BALB/c mice were administered 34.1 g crude drug/kg YPFS intragastrically twice daily for 3 days, and normal saline was administered as a control (control serum). One hour after the final administration, blood was collected and centrifuged at 2200 g for 15 min after standing for 1 h. The serum was filtered through a 0.22 µm nylon filter membrane and stored at −80°C. Serum from YPFS-treated mice was used to evaluate the effect of YPFS on TNF-α induced TSLP production in 16HBE cells.

Part of the serum from YPFS-treated mice (200 µL) was mixed with ethyl acetate (400 µL), vortexed for 3 min, and centrifuged at 1600 g for 5 min. Supernatants were collected and dried under nitrogen at 45°C. Residues were dissolved in 200 µL methanol and centrifuged at 10000 g for 5 min and filtered through a 0.45 µm nylon membrane filter before HPLC-MS analysis.

### HPLC-MS analysis

Analysis were performed on an Waters ZQ2000 LC-MS system equipped with a quaternary gradient pump, an autosampler, and a column incubator, a diode-array detector (DAD) and an electrospray ionization (ESI) ion source, connect to Masslynx 4.0 workstation. A chromatographic column (4.6×250 mm, 5µm; Chrom-Matrix) was used. The liquid phase conditions were (A) acetonitrile (Tedia, USA); (B) water:formic acid (100∶0.05, v/v). The flow rate was 1 mL/min and elution conditions were: 0–10 min, linear gradient 3%–15% (v/v) A in B; 10–20 min, linear gradient 15%–20% A; 20–30 min, linear gradient 20%–28% A; 30–40 min, linear gradient 28%–40% A; 40–60 min, linear gradient 40%–60% A; 60–85 min, 60%–95% A isocratic; 85–95 min, linear gradient 95%–3% A. The system operated at 30°C and the injection volume was 10 µL. The DAD was set to scan from 200 to 400 nm.

The mass spectrometry conditions were: capillary voltage 3 kV, cone voltage 40 V, source temperature 120°C, desolvation temperature 400°C, desolvation gas flow 400 L/h, cone gas flow 50 L/h. The ESI was performed in the positive and negative ionization modes in the scan range 100–1000 nm.

### Luciferase reporter assay *in virto*

Functional nuclear factor (NF) -κB activation was determined by luciferase reporter assay. 16HBE cells were seeded at a density of 1×10^5^cells/mL in 24-well plates and transfected with pNFκB-TA-luc plasmid (Beyotime Biotechnology, Haimen, China) containing the response element that drives transcription of the luciferase reporter gene, along with Renilla using Lipofectamine TM 2000 (Invitrogen Carlsbad, CA) according to the manufacturer's instructions for 6 h. Cells were further incubated with fresh medium for 24 h. Cells were pretreated with different concentrations of claycosin or formononetin for 2 h or with culture medium as a control before stimulated with TNF-α (100 ng/mL) for 1 h. The luciferase activity was measured in the cellular extracts using a dual luciferase reporter assay system (Promega, Madison, WI, USA) with GloMax 20/20 n Luminometer (Promega).

### Immunofluorescence assay *in virto*

NF-κB nuclear translocation was evaluated by immunofluorescence assay. 16HBE cells were starved with serum-free medium overnight and were pretreated with claycosin or formononetin for 2 h or with culture medium as a control before stimulated with TNF-α (100 ng/mL) for 1 h. The cells were fixed in ice-cold paraformaldehyde (PFA) for 1 h. The coverslips were washed with PBS before permeabilizing the cells with Triton X-100 (Genview, Scientific Inc, USA) and blocked with 5% bovine serum albumin (BSA) for 1 h at 37°C. The cells were probed with 4 µg/mL rabbit monoclonal antibody to NF-κB (Santa Cruz Biotechnology, Santa Cruz, CA, USA) at 4°C overnight. After repeated washes with PBS, the cells were probed with 1 µg/mL goat anti-rabbit IgG conjugated to FITC (Santa Cruz Biotechnology) and 4′,6-diamidino-2-phenylindole (DAPI, Santa Cruz Biotechnology) at a concentration of 5 µg/mL for 1 h. The labeled sections were viewed with fluorescence confocal microscopy (Olympus, Tokyo, Japan).

### Cytotoxicity assay *in vitro*

Cytotoxicity was determined with the methyl tetrazolium (MTT) assay. Cells were seeded in 96-well plates (1×10^4^ cells/well) and were incubated for 12 or 24 h in the presence of indicated doses of calycosin and formononetin. 20 µL MTT (5 mg/mL; Sigma) stock solution was added to each well, and plates were incubated at 37°C. After 4 h incubation, cells were lysed with dimethyl sulfoxide (DMSO, Sigma). Absorbance was measured at 490 nm using Synergy HT Multi-Mode Microplate Reader (Bio-Tek, Winooski, VT, USA). All experiments were performed in triplicate.

### Experimental ACD model *in vivo*

To establish the ACD model, BALB/c mice were topically sensitized with 1.5% FITC solution on the abdominal skin on days 1 and 2 and elicited on the right ear with 0.5% FITC solution on day 6. Mice were treated once daily with calycosin (0.5, 5 or 10 mg/kg, intraperitoneally), formononetin (0.5, 5 or 10 mg/kg, intraperitoneally) or normal saline 2 days before sensitization until day 3 of the model (only administrated at initiation stage of sensitization phase). Ear thickness was measured 24 h after elicitation (Harbin Measuring & Cutting Tool Group Co. Ltd. Harbin, China) and changes in ear thickness were calculated. Histopathological changes in the ears were examined by hematoxylin and eosin (H&E) staining.

### Statistical analysis

The data were expressed as means ± SD. Multiple groups' comparisons were analyzed by one-way analysis of variance, and Dunnett's test was used for comparison between two groups, with GraphPad Prism 5 (GraphPad Software, San Diego, CA, USA). Statistical significance was set at p<0.05.

## Results

### 1 Effect of YPFS on TSLP production *in vivo* and *in vitro*

#### 1.1 YPFS inhibited TSLP production at the initial stage of sensitization *in vivo*

**Figure 1:**
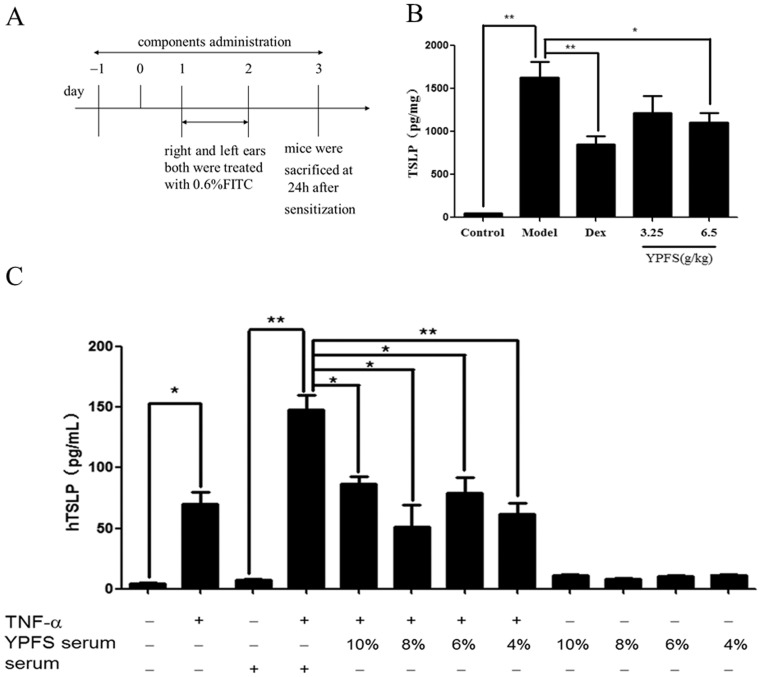
Effect of YPFS on TSLP *in vivo* and *in vitro*. (A) Flow charts of the TSLP production model. (B) Effect of YPFS on the production of TSLP in ear tissue at the initial stage in a murine model of ACD. TSLP in ear homogenates was analyzed by ELISA and total protein were examined by BCA kit. TSLP level was assessed with the formula: concentration of TSLP in the homogenate/total protein (pg/mg). (mean±SD, n = 8, *p<0.05, **p<0.01). (C) Effect of YPFS on TSLP production induced by TNF-α in 16HBE cells. 16HBE cells were treated with 1.25 µg/mL TNF-α and YPFS serum (4%, 6%, 8% or 10%) or control serum for 12 h. TSLP protein level was analyzed by ELISA. (mean±SD, n = 3, *p<0.05, **p<0.01) All the experiments were performed in triplicates.

A murine model of TSLP production at the initial stage of ACD was established to observe the effect of YPFS on TSLP production *in vivo*. Mice were treated once daily with 3.25 or 6.5 g/kg YPFS, intragastrically, or 0.67 mg/kg dexamethasone, intraperitoneally, 2 days before treatment with FITC until day 3 of the model (Fig. 1A). The level of TSLP was significantly higher in the model compared with the control group. YPFS (6.5 g/kg) decreased the level of TSLP in this model (Fig. 1B). The result implies that YPFS might affect allergic sensitization process by regulating TSLP.

#### 1.2 TSLP production in 16HBE cells was reduced by serum from YPFS-treated mice *in vitro*

We studied the effect of serum from YPFS-treated mice on TNF-α-induced TSLP production in 16HBE cells. Serum pharmacology is a widely used method to evaluate effects of Chinese medicine *in vitro* to exclude the disturbance of irrelevant factors, such as pH, osmolality, ion and so on [28]. Cells were treated with 4%–10% serum from YPFS-treated mice and 1.25 μg/mL TNF-α simultaneously for 12 h. Serum from YPFS-treated mice did not alter the TSLP basal levels when there was no stimulation. A substantial amount of TSLP was detected in the supernatant following stimulation of 16HBE cells with 1.25 μg/mL TNF-α. Serum from YPFS-treated mice significantly decreased TSLP level in the presence of TNF-α (Fig. 1C). These results were consistent with previous observations *in vivo* and implied that regulation of TSLP might be an important mechanism of YPFS.

### 2 Probing potential active components of YPFS by 16HBE cell binding combined with HPLS-MS (16HBE-HPLC-MS)

#### 2.1 HPLC fingerprint of YPFS extract

**Figure 2:**
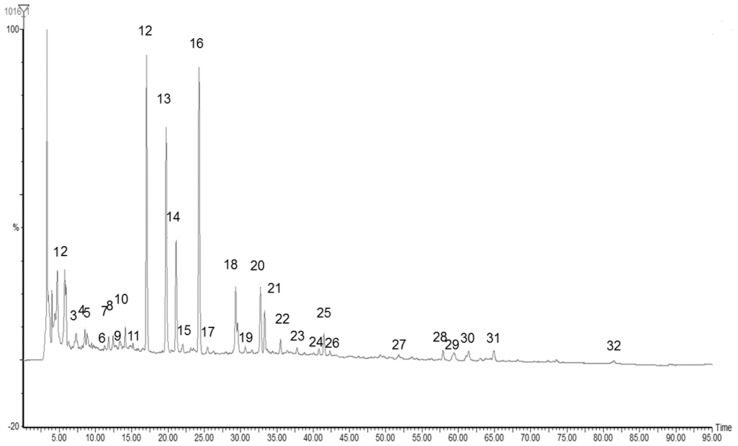
HPLC fingerprint of YPFS extract at 254 nm. YPFS was detected by HPLC-MS with an optimal condition for obtaining maximum peaks. There were 32 main peaks in YPFS extract. All the experiments were performed in triplicates.

We performed 16HBE-HPLC-MS to screen for active components of YPFS that interacted with ECs. We established the fingerprint of YPFS as the background information for 16HBE-HPLC-MS. There were 32 main peaks in the fingerprint of YPFS extract at 254 nm (Fig. 2). Among these, eight components were identified after comparison with the standard materials (Table 1). All of the main peaks were well separated.

**Table 1:** RRT and RPA of each peak in fingerprint of YPFS extract.

No.	RRT	RPA	Identified compound
1	0.195039	0.409416	
2	0.237648	0.4078	
3	0.301521	0.077928	
4	0.35163	0.056677	
5	0.363823	0.0987593	
6	0.463535	0.0121612	
7	0.486958	0.039583	
8	0.511683	0.041818	
9	0.54162	0.037255	
10	0.581036	0.047355	
11	0.625051	0.031994	
12	0.7019	0.843082	Prim-O-glucosylcimifugin
13	0.813162	0.766692	Calycosin-7-glucoside copyranoside
14	0.869494	0.394774	Cimifugin
15	0.906581	0.035079	
16	1	1	4′-O-glucopyranosyl-5-O-methylvisamminol
17	1.048749	0.02977	
18	1.208102	0.367158	Ononin
19	1.263032	0.025007	
20	1.348869	0.247059	Claycosin
21	1.372934	0.129639	Sec-o-glucosylhamaudol
22	1.462892	0.047043	
23	1.55697	0.021852	
24	1.681296	0.020301	
25	1.710141	0.082697	Formononetin
26	1.743808	0.018922	
27	2.134322	0.017997	
28	2.386657	0.038159	
29	2.448469	0.067835	
30	2.531545	0.056232	
31	2.675114	0.04355	
32	3.351017	0.02101	

The injection precision was determined by five replicate injections of the same sample in one day. The relative standard deviations (RSDs) of relative retention time (RRT) and relative peak area (RPA) were 0.01%–0.32% and 0.28%–2.83%, respectively, and there were no significant changes. The repeatability was assessed by analyzing five independently prepared samples of YPFS extract. The RSDs of RRT and RPA were 0.01%–0.28% and 0.15%–2.88%, respectively, and there were no significant changes. The sample stability was assessed by successive injections of the same sample at 0, 3, 6, 9, 12 and 24 h. During this period, the solution was stored at room temperature. The RSDs of RRT and RPA were 0.04%–0.71% and 0.42%–2.81%, respectively, and there were no significant changes. The results of injection precision, repeatability and stability indicated that this method was adequate, valid and applicable. The RRT and RPA of the 32 peaks are shown in Table 1.

#### 2.2 Probing 16HBE-binding components of YPFS

**Figure 3:**
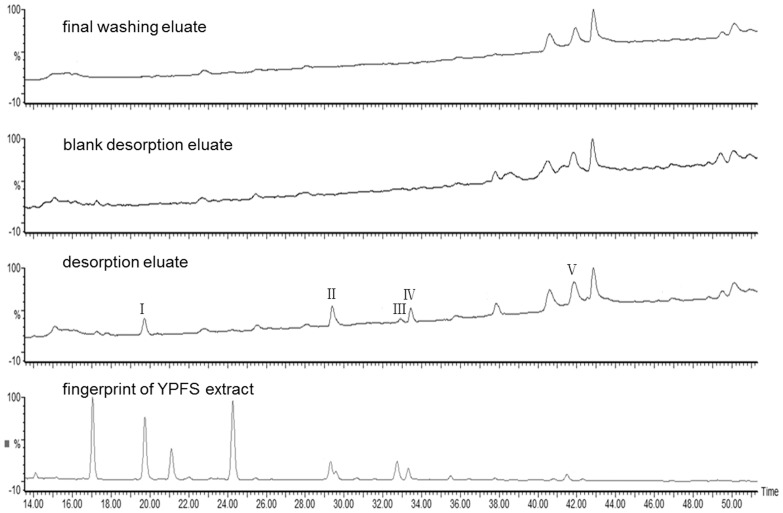
Comparative chromatograms of fingerprint of YPFS extract, final wash eluate, blank desorption eluate (in which YPFS extract was replaced by RPMI 1640) and 16HBE-binding desorption eluate. Detection was at 254 nm. All the experiments were performed in triplicates.

We selected 16HBE cells to explore the potential active components of YPFS. Five principal peaks (I–V) were detected at 254 nm in the YPFS 16HBE-binding desorption eluate, and no comparable peaks were detected in the chromatograms of the two control samples (final wash and blank desorption eluates) (Fig. 3). All the captured peaks were also found in the fingerprint of YPFS, which indicated that these components were from YPFS.

#### 2.3 Identification of chemicals binding to 16HBE cells by HPLC-MS

**Figure 4:**
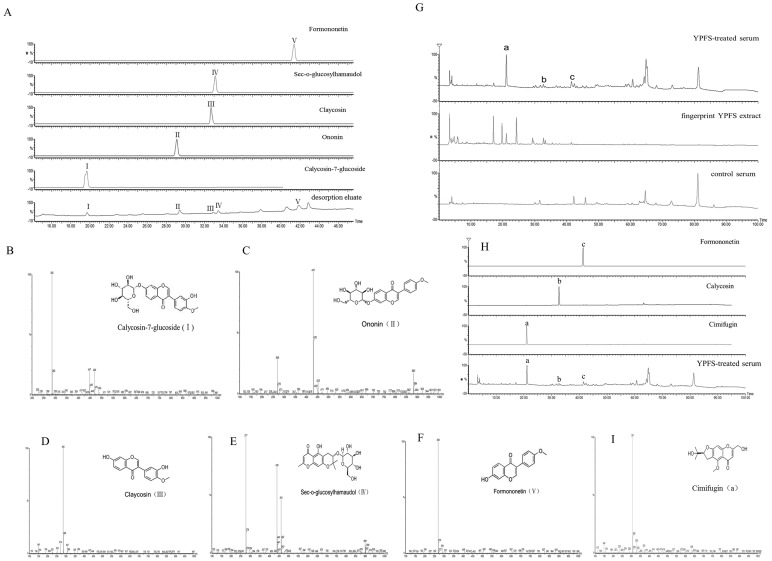
Determination of components in serum from YPFS-treated mice and identification of YPFS extract chemicals binding to 16HBE cells by HPLC-MS. (A) UV visible spectrum of YPFS components I–V in the 16HBE-binding desorption eluate corresponding to the standard materials at 254 nm. (B) ESI-MS negative ionization spectrum of compound I and its structural formula (inset). Calycosin-7-glucoside, (+)ESI-MS m/z was 447[M+H]^+^(m/z). (C) ESI-MS negative ionization spectrum of compound II and its structural formula (inset). Ononin, (+)ESI-MS m/z was 431[M+H]^+^(m/z). (D) ESI-MS negative ionization spectrum of compound III and its structural formula (inset). Claycosin, (+)ESI-MS m/z was 285[M+H]^+^(m/z). (E) ESI-MS negative ionization spectrum of compound IV and its structural formula (inset). Sec-o-glucosylhamaudol, (+)ESI-MS m/z was 439[M+H]^+^(m/z). (F) ESI-MS negative ionization spectrum of compound V and its structural formula (inset). Formononetin, (+)ESI-MS m/z was 269[M+H]^+^(m/z). (G) UV spectrum of serum from YPFS-treated mice, compared with fingerprint of YPFS extract and control serum at 254 nm. (H) UV spectrum of components a–c detected in serum of YPFS-treated mice corresponding to the standard materials at 254 nm. (I) ESI-MS negative ionization spectrum of compound “a” and its structural formula (inset). Cimifugin, (+)ESI-MS m/z was 307[M+H]^+^(m/z). All the experiments were performed in triplicates.

To identify the above chemicals, we analyzed the molecular weight by MS and compared it with reference values [29]. Retention time and UV spectrum of the five peaks in HPLC were corresponded with the standard materials (Fig. 4A). In negative ion mode, the product ion [M+H]^+^ at m/z 447 was calycosin-7-glucoside; the transition m/z 447→285 was chosen. The subtraction of the two ions was m/z 162 (neutral loss glucose). The product ion [M+H]^+^at m/z 431was ononin; the transition m/z 431→269 was chosen. The subtraction of the two ions was m/z 162 (neutral loss glucose). The product ion [M+H]^+^at m/z 285 was claycosin. The product ion [M+H]^+^ at m/z 439 was sec-o-glucosylhamaudol; the transition m/z 439→277 was chosen. The product ion [M+H]^+^ at m/z 269 was formononetin (Fig. 4B–F). In conclusion, the five peaks were identified as calycosin-7-glucoside (I), ononin (II), claycosin (III), sec-o-glucosylhamaudol (IV) and formononetin (V) (Table 2).

**Table 2:** Peak assignment for the components from desorption eluate.

Peak no.	Compound name	t_R_(min)	(+)ESI-MS m/z	Other ions	UV λ_max_(nm)
I	Calycosin-7-glucoside	19.73	447[M+H]^+^(m/z)	285	260,290
II	Ononin	29.40	431[M+H]^+^(m/z)	269	255,301
III(b)	Claycosin	32.90	285[M+H]^+^(m/z)	-	250,290
IV	Sec-o-glucosylhamaudol	33.45	439[M+H]^+^(m/z)	277	250,298
V(c)	Formononetin	41.87	269[M+H]^+^(m/z)	-	250,303
a	Cimifugin	20.99	307[M+H]^+^(m/z)	-	216,229

#### 2.4 Analysis of components in serum from YPFS-treated mice

Generally, the components of Chinese medicine need to be absorbed into the blood to exert their effects. Therefore, serum from YPFS-treated mice was analyzed to establish which components were present in blood. Three main peaks (a–c) were detectable in serum from YPFS-treated mice, as opposed to none in control serum (Fig. 4G). These peaks were also found in the fingerprint of YPFS. Retention time and UV spectrum of the three peaks in HPLC corresponded with the standard materials (Fig. 4H). In negative ion mode, the product ion [M+H]^+^ at m/z 285 was claycosin (Fig. 4D). The product ion [M+H]^+^ at m/z 285 was formononetin (Fig. 4F). The product ion [M+H]^+^ at m/z 307 was cimifugin (Fig. 4I). Consequently, the major components in serum from YPFS-treated mice were claycosin, formononetin and cimifugin.

Among these components, claycosin and formononetin were both detected by 16HBE-HPLC-MS and analysis of serum from YPFS-treated mice. It is reasonable to speculate that these two compounds are the most likely to be active components in YPFS. Claycosin and formononetin were studied to explore further their effect on regulating TSLP and allergic inflammation.

### 3 Claycosin and formononetin are effective in regulating TSLP and attenuating allergic inflammation

#### 3.1 Claycosin and formononetin reduced TSLP production at the initial stage of allergic inflammation *in vivo*

**Figure 5:**
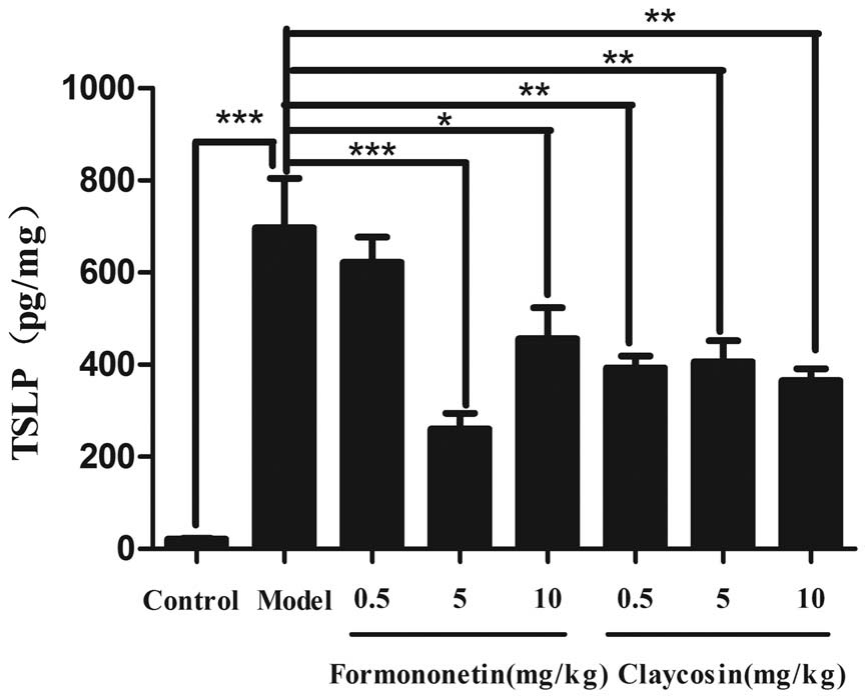
Effect of claycosin and formononetin on the production of TSLP at the initial stage in a murine model of ACD *in vivo*. TSLP in ear homogenates was analyzed by ELISA and total protein was determined by BCA kit. TSLP level was assessed with the formula: concentration of TSLP in homogenate/total protein (pg/mg). (mean±SD, n = 8, *p<0.05, **p<0.01, ***p<0.001). All the experiments were performed in triplicates.

To verify the effects of these two compounds *in vivo*, a TSLP production model at the initial stage of allergic inflammation was utilized. Mice were treated once daily with formononetin and claycosin (0.5, 5 or 10 mg/kg, intraperitoneally) or vehicle 2 days before treatment with FITC, until day 3 of the model. After FITC treatment, mice exhibited increased TSLP production in the ears. TSLP levels in the ear homogenates were reduced markedly by formononetin (5 mg/kg) and claycosin (0.5, 5 and 10 mg/kg) (Fig. 5), which indicated that these two compounds could inhibit TSLP production in the initial stage of allergic inflammation.

#### 3.2 Claycosin and formononetin suppressed transcriptional activation of NF-κB induced by TNF-α *in vitro*

**Figure 6:**
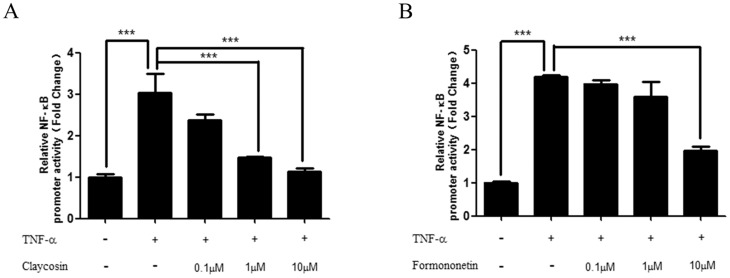
Effect of claycosin and formononetin on the transcriptional activation of NF-κB induced by TNF-α. 16HBE cells transfected with pNFκB-TA-luc were pretreated with claycosin or formononetin for 2 h or treated with medium only as a control, then stimulated with TNF-α(100 ng/mL) for 1 h. Transcriptional activation of NF-κB was measured in the cellular extracts using a dual luciferase reported gene assay kit. (A) Effect of claycosin on the transcriptional activation of NF-κB. (B) Effect of formononetin on the transcriptional activation of NF-κB. (mean±SD, n = 3, ***p<0.001). All the experiments were performed in triplicates.

We explored whether claycosin and formononetin affected NF-κB signaling in 16HBE cells. After transfection with pNFκB-TA-luc plasmid, 16HBE cells were pretreated with different concentrations of claycosin or formononetin for 2 h or with culture medium as a control before TNF-α stimulation. NF-κB transcriptional activity was strongly induced after stimulation of transfected cells with TNF-α (100 ng/mL) for 1 h. The transcriptional activity of NF-κB was significantly reduced in cells treated with calycosin (1 and 10 µM) (Fig. 6A) and formononetin (10 µM) (Fig. 6B). The results indicated that calycosin and formononetin were able to inhibit NF-κB transcriptional activity, which might subsequently suppress the production of TSLP.

#### 3.3 Claycosin and formononetin restricted NF-κB translocation *in vitro*

**Figure 7:**
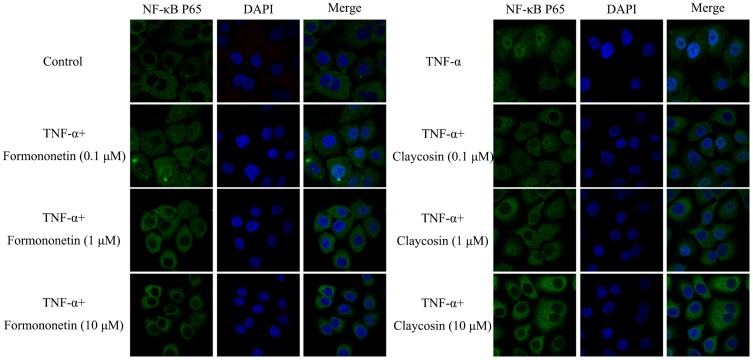
Effect of claycosin and formononetin on NF-κB translocation. NF-κB translocation in 16HBE cells was examined by immunofluorescence assay. n = 3. All the experiments were performed in triplicates.

The above studies showed that claycosin and formononetin downregulated transcriptional activation of NF-κB *in vitro* and reduced TSLP production *in vivo*. However, it was unclear whether these two compounds affected nuclear translocation of NF-κB. Translocation of NF-κB was analyzed in 16HBE cells, which were pretreated with claycosin or formononetin for 2 h or with culture medium as a control before stimulation. After 1 h stimulation of the 16HBE cells with 100 ng/mL TNF-α, immunofluorescence revealed that NF-κB (p65) was predominantly present in the nucleus, compared to the control cells. Formononetin and claycosin at 0.1 µM partially inhibited translocation of NF-κB to the nucleus. More obviously, formononetin and claycosin at 1 and 10 µM significantly suppressed NF-κB nuclear translocation, wherein NF-κB (p65) was predominantly present in the cytosol (Fig. 7). Hence, formononetin and claycosin downregulation of TSLP expression might be due to blocking nuclear translocation of NF-κB.

#### 3.4 Evaluation of claycosin and formononetin cytotoxicity in 16HBE cells

**Figure 8:**
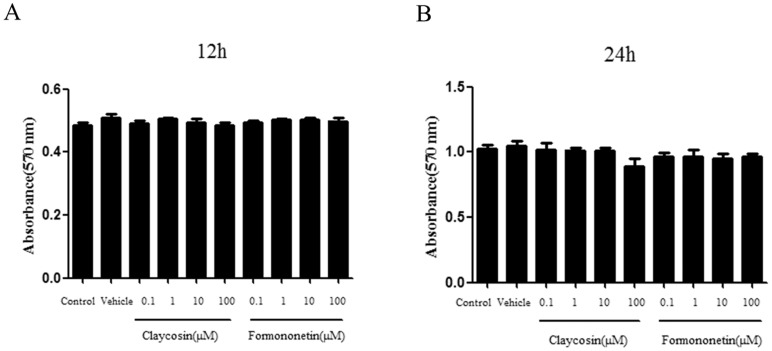
Effect of claycosin and formononetin on 16HBE cell proliferation. (A) Effect of claycosin and formononetin on 16HBE cell proliferation for 12 h. (B) Effect of claycosin and formononetin on 16HBE cell proliferation for 24 h. (mean±SD, n = 3). All the experiments were performed in triplicates.

We studied the effect of claycosin and formononetin on cytotoxicity of 16HBE cells by modified MTT assay. 16HBE cells were incubated with different concentrations (0.1–100 μM) of these compounds for 12 and 24 h. Vehicle control groups were treated with DMSO (0.01%, v/v; Sigma) and control groups were treated with RPMI 1640. Claycosin and formononetin had no significant effect on 16HBE proliferation at 12 h (Fig. 8A) or 24 h (Fig. 8B). Thus, their effect on TSLP and NF-κB should not be attributed to cytotoxicity.

#### 3.5 Allergic inflammation was attenuated by calycosin and formononetin administered only at the initial stage of sensitization

Calycosin and formononetin had a significant effect on TSLP at the initial stage of allergic inflammation in this study, and affected transcriptional activation and translocation of NF-κB. Nevertheless, are these effects sufficient to interfere with allergic inflammation? If yes, administering the compounds at the initial stage of the sensitization phase in the ACD model would suppress eventual inflammation.

**Figure 9:**
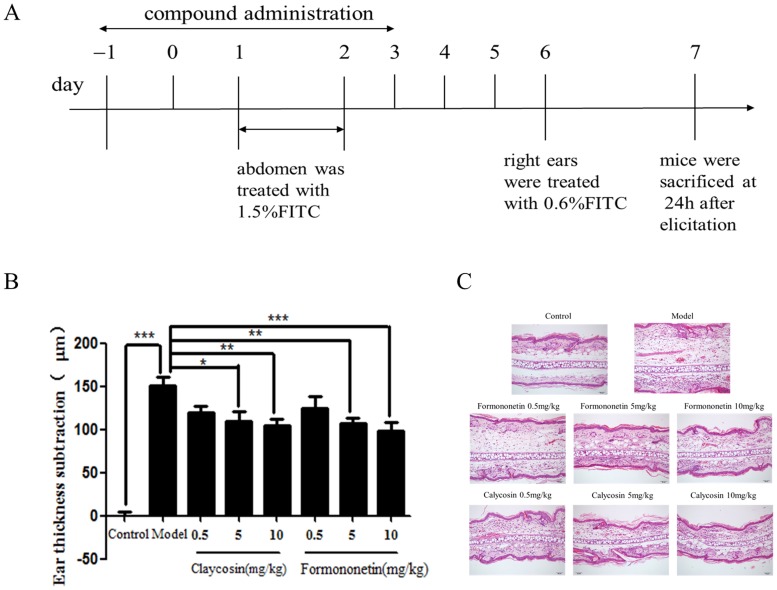
Effect of calycosin and formononetin administered at the initial stage of sensitization on ear thickness and histopathological changes in ACD mice. (A) Flow charts of compound administration in ACD murine model. (B) Effect of calycosin and formononetin on ear thickness. (mean±SD, n = 8, *p<0.05, **p<0.01, ***p<0.001). (B) Histopathological changes of dermatitis examined by H&E staining. All the experiments were performed in triplicates.

Mice were treated once daily with calycosin (0.5, 5 or 10 mg/kg, intraperitoneally), formononetin (0.5, 5 or 10 mg/kg, intraperitoneally) or normal saline 2 days before sensitization until day 3 of the model (only administrated at initiation stage of sensitization phase, Fig. 9A). Ear thickness of ACD mice was significantly decreased by treatment with the two compounds (Fig. 9B). In terms of pathological changes, thickening of the epidermis and infiltration of inflammatory cells were evident compared with the normal ear in control mice. After treatment with calycosin and formononetin, the thickening of epidermis and infiltration of inflammatory cells were alleviated (Fig. 9C). These results indicated that the effects of calycosin and formononetin at the initial stage of sensitization are sufficient to suppress the eventual allergic inflammation in the ACD model.

## Discussion

YPFS affected TCR and MHC class II expression on CD4^+^ T cells in a mouse model of asthma, and regulated the balance of Th1/Th2 cells in murine allergic airway disease [6]. However, the relevant mechanism underlying these functions has not yet been identified. TSLP derived from ECs is a key allergic inflammation master switch [20], [21], [30], suggesting that it is the most important target interfering with the initial phase of allergic diseases [31]–[33]. Most studies have shown that epithelial cells are the main source of TSLP production [34]–[36]. These results directly confirm the link between TSLP and allergic inflammation. In this study, we reported that YPFS significantly inhibited TSLP production *in vivo* and serum from YPFS-treated mice decreased TSLP production *in vitro*, which indicated that regulating TSLP is the underlying mechanism of YPFS in reducing allergy relapse.

Although both clinical and experimental studies attest to the prophylactic and/or therapeutic efficacy of YPFS, the bioactive molecules have not yet been identified. Previous studies have suggested that purified YPFS polysaccharides have immunostimulatory activity and represent the active components of YPFS [37]. However, the molecules responsible for the immunosuppressive effect of YPFS have not been identified. The active molecules are inferred to interact with target cells, therefore, cell-based affinity purification and assay techniques have been used as a screener to capture the bioactive components in Chinese medicines. Our group has established the cell-binding methods to screen for the potential active components in Chinese medicine including hepatocyte [22], [23] and erythrocyte [24] binding. Here, we used ECs to establish a method called 16HBE-HPLC-MS to capture the potential bioactive components. Five components were found to bind to 16HBE cells. They were identified as calycosin-7-glucoside, ononin, claycosin, sec-o-glucosylhamaudol and formononetin. We analyzed serum from YPFS-treated mice and three main components were detected. Claycosin and formononetin were both detected by 16HBE-HPLC-MS and in serum from YPFS-treated mice. This indicated that these two compounds were most likely to be the potential active components in YPFS.

There are some reports about the bioactivities of these compounds. Calycosin at low concentrations promotes the proliferation of MCF-7 cells and inhibits apoptosis [38]. Formononetin has estrogen-like effects in the body that improve dyslipidemia in high-fat diet rats [39], and inhibits migration and proliferation of vascular smooth muscle cells induced by platelet-derived growth factor BB [40]. However, these results had no direct relationship with inhibition of relapse of allergic diseases. Our data suggested that TSLP level in the ears was significantly reduced by claycosin and formononetin at the initial stage in a murine model of TSLP production. NF-κB signaling is essential for epithelial production of TSLP, thus, we assessed the transcriptional activity and translocation of NF-κB to explore the effect of these compounds. Calycosin and formononetin reduced the transcriptional activity of NF-κB and blocked its nuclear translocation. The results implied that these compounds might be active in inhibiting TSLP through regulating NF-κB, consequently contributing to the antiallergic effect of YPFS.

We found that claycosin and formononetin administered only at the initial stage of sensitization attenuated thickening of the epidermis and decreased infiltration of inflammatory cells in the ear of the ACD model. These results indicate that effects of calycosin and formononetin at the initial stage of sensitization are sufficient for suppression of allergic inflammation. Therefore, regulation of TSLP might be the mechanism for this effect on allergic inflammation. We will explore further the signaling pathway and the mechanisms of these detected components for regulating TSLP.

In summary, EC binding combined with HPLC-MS is a valid method for screening active components from complex mixtures of Chinese medicine. The compounds screened from YPFS alleviated allergic inflammation by inhibiting TSLP through regulating NF-κB activation and translocation. These components might have important implications for screening promising drugs in allergic diseases.
